# City-scale monitoring of antibiotic resistance genes by digital PCR and metagenomics

**DOI:** 10.1186/s40793-024-00557-6

**Published:** 2024-03-15

**Authors:** Lucia Maestre-Carballa, Vicente Navarro-López, Manuel Martinez-Garcia

**Affiliations:** 1 Department of Physiology, Genetics, and Microbiology, University of Alicante, Carretera San Vicente del Raspeig, San Vicente del Raspeig, Alicante, 03690 Spain; 2 Instituto Multidisciplinar para el Estudio del Medio Ramon Margalef, University of Alicante, San Vicente del Raspeig, Alicante, 03690 Spain; 3 Clinical Microbiology and Infectious Disease Unit, Hospital Universitario Vinalopó, Elche, Spain

**Keywords:** Antibiotic resistance gene, Metagenomics, Digital PCR, DPCR, ARG, Bacteria, Virus, Wastewater, *sul2*, *tetW*, Resistome

## Abstract

**Background:**

Anthropogenic activities significantly contribute to the dissemination of antibiotic resistance genes (ARGs), posing a substantial threat to humankind. The development of methods that allow robust ARG surveillance is a long-standing challenge. Here, we use city-scale monitoring of ARGs by using two of the most promising cutting-edge technologies, digital PCR (dPCR) and metagenomics.

**Methods:**

ARG hot-spots were sampled from the urban water and wastewater distribution systems. Metagenomics was used to provide a broad view of ARG relative abundance and richness in the prokaryotic and viral fractions. From the city-core ARGs in all samples, the worldwide dispersed *sul2* and *tetW* conferring resistance to sulfonamide and tetracycline, respectively, were monitored by dPCR and metagenomics.

**Results:**

The largest relative overall ARG abundance and richness were detected in the hospital wastewater and the WWTP inlet (up to ≈6,000 ARGs/Gb metagenome) with a large fraction of unclassified resistant bacteria. The abundance of ARGs in DNA and RNA contigs classified as viruses was notably lower, demonstrating a reduction of up to three orders of magnitude compared to contigs associated to prokaryotes. By metagenomics and dPCR, a similar abundance tendency of *sul2* and *tetW* was obtained, with higher abundances in hospital wastewater and WWTP input (≈125–225 ARGs/Gb metagenome). dPCR absolute abundances were between 6,000 and 18,600 copies per ng of sewage DNA (≈10^5–7^ copies/mL) and 6.8 copies/mL in seawater near the WWTP discharging point.

**Conclusions:**

dPCR was more sensitive and accurate, while metagenomics provided broader coverage of ARG detection. While desirable, a reliable correlation of dPCR absolute abundance units into metagenomic relative abundance units was not obtained here (r^2^ < 0.4) suggesting methodological factors that introduce variability. Evolutionary pressure does not significantly select the targeted ARGs in natural aquatic environments.

**Supplementary Information:**

The online version contains supplementary material available at 10.1186/s40793-024-00557-6.

## Background

In modern medicine, the advent of antibiotics is one of the most remarkable achievements, revolutionizing the treatment of bacterial infections and saving countless lives. However, the emergence and dissemination of antibiotic resistant bacteria pose a grave and escalating threat to global public health, challenging the efficacy of antibiotics [34]. Antibiotic resistance, characterized by the ability of bacteria to withstand the lethal effects of antibiotics, represents a multifaceted challenge of monumental proportions that will result in an annual death toll of 10 million by 2050 [1]. The main mechanisms of resistance are: limiting uptake of a drug, modification of a drug target, inactivation of a drug, and active efflux of a drug [36]. These mechanisms may be native to microorganisms or genetically acquired from other microorganisms via horizontal gene transfer.

Antibiotic Resistance Genes (ARGs) can emerge virtually anywhere in the world and can be spread through various means, such as water, food or air, and at different transference rates [27]. ARGs have been detected in natural environments (e.g. aquatic, soil, and air), engineered and clinical habitats [45], and human microbiome [25]. Anthropogenic activities, including the clinical use and abuse of antibiotics and farming are widely regarded as the main drivers of ARGs dissemination [45]. Multiple examples of ARGs, such as the New Delhi metallo-beta-lactamase genes, have emerged clinically and rapidly disseminated worldwide [21]. Unfortunately, there are many examples like that, and recently a large metagenomic study has demonstrated that 25% of the detected ARGs in various habitats, pose an evident health risk [45]. Unraveling the intricate web of ARGs, elucidating their genetic architecture, dissemination mechanisms, and settling methods for properly monitoring ARG dispersion are fundamental pursuits for correct global surveillance. For instance, metagenomics, and in particular sewage metagenomics, has been proposed as a convenient method for ARG monitoring to determine the diversity and abundance [14, 32]. Surveillance solutions based on shotgun metagenomic sequencing have the advantage of being relatively hypothesis-agnostic albeit well-settled international metagenomic procedure standards (i.e. experimental and bioinformatic analysis) are clearly lacking, but under consideration in different fields [20]. To the best of our knowledge, the only example in ARG monitoring is the recent ISO-certified bioinformatic workflow for the identification and surveillance of ARGs from bacterial genomic data from isolates, which was compared and validated with PCR and quantitative PCR [38], methodology which allows to determine the amount of product in real-time. Undoubtedly, PCR-based surveillance methods have been shown to be highly robust, useful, consistent, and universally standardized for detecting hallmark genes (e.g. SARS-CoV-2; ISO norm ISO/TS 5798:2022). For instance, the European Reference Laboratory for Antimicrobial Resistance published a list of available and validated primers for monitoring ARGs (https://www.eurl-ar.eu/). Among the different PCR techniques, digital PCR provides absolute gene quantification and surpasses the precision of qPCR with much higher sensitivity and precision, without the need for a calibration curve. Recently, dPCR technologies have been implemented to quantify the mobility of ARGs [8]. Consequently, methods for quantitatively assessing the potential mobility of ARGs that are easily applicable and of low cost are urgently needed, and thus the urgent need for a conclusive global framework addressing this issue in environmental samples is beyond dispute [8].

Here, we employ a city-scale distribution of waterborne ARGs by using two of the most promising cutting-edge technologies for ARG surveillance: metagenomics and digital PCR data from environmental samples, focusing on genes involved in tetracycline and sulfonamide resistance and balancing broad coverage and high sensitivity. By using metagenomics, we first monitored the diversity and abundance of ARGs throughout the whole water system in Alicante city (≈331,000 people, Spain) including five sampling locations: untreated drinking water that feeds Alicante City, wastewater from the largest hospital, input and output from one of the largest wastewater treatment plants (WWTP) in Alicante city, and a seawater sample taken nearby (dozens of meters) to the mouth of the WWTP that discharges the treated wastewater to the Mediterranean Sea. In addition to monitoring ARGs in the prokaryotic fraction, we also considered the viral fraction in some of the samples. Then, from the list of detected ARGs in all samples (core ARGs), we subsequently selected the genes *sul2* and *tetW* to be closely monitored by digital PCR and metagenomics. These genes confer resistance to sulfonamide and tetracycline antibiotics respectively, and are commonly detected in sewage and associated with the ‘farm to fork’ [44]. In addition, the *sul2* gene is highly mobilized by plasmids [17], and for instance, *tetW* gene was ranked among the top 15 ARGs most frequently found in 79 wastewater samples analyzed from 60 different countries and 5 continents [14].

Thus, our study represents one of the first side-by-side comparisons of metagenomic and dPCR data from representative urban samples in line with the One Health strategy. Although it does not aim to settle the debate about the best strategy to follow, which requires a large collaborative translational effort, our study provides valuable insights that aid in discussing the pros and cons of each technology in the real context of a medium-sized city.

## Materials and methods

### Sampling spots

**Fig. 1:**
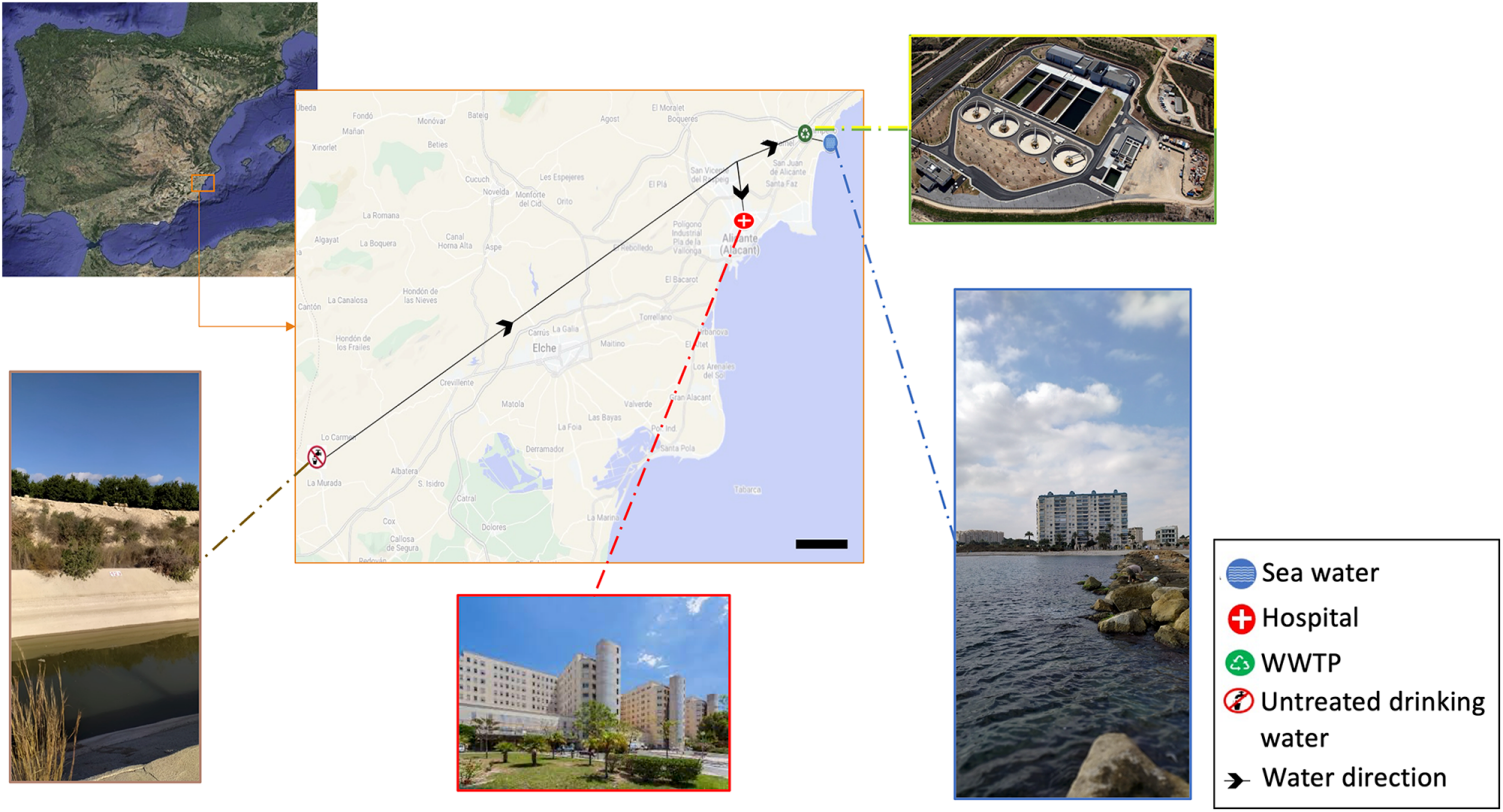
Sampling locations in Alicante city used in this study. Water samples were collected from various locations in the city of Alicante considered as hot spots for ARGs dispersion. Water samples were obtained from untreated drinking water of the Crevillente’s channel (38°11’23.4"N, 0°58’13.5"W), wastewater of the Hospital General Universitario Dr. Balmis, (38°21’47.9"N, 0°29’08.6"W) and from the wastewater treatment plant (WWTP) l’Alacantí Nord (38°25’38.5"N, 0°25’03.5"W) and seawater (38°25’08.6"N, 0°23’16.7"W) near the outlet of the mentioned WWTP. Picture obtained from Google Maps. Scale bar: 5 km

Different hot spots of the city of Alicante (331,000 citizens) were sampled (Fig. 1). The following water samples were analyzed: (1) Untreated drinking water from one of the main water channel of Crevillente (38°11’23.4"N, 0°58’13.5"W; 11/20/20) that feed Alicante city, (2) Wastewater samples from the largest hospital in Alicante (Hospital General Universitario Dr. Balmis, 38°21’47.9"N, 0°29’08.6"W; 05/14/19), (3) the inlet of the WWTP l’Alacantí Nord, which receives municipal wastewater, from now on “WWTP input”, (38°25’30.8"N, 0°25’10.1"W; 03/26/19, 01/28/20 and 02/19/20), (4) the output or treated wastewater by the aforementioned WWTP (38°25’38.5"N, 0°25’03.5"W; 05/11/16, 01/02/20 and 02/19/20) and (5) seawater obtained in a spot nearby the WWTP outlet placed in Campello (38°25’08.6"N, 0°23’16.7"W; 02/27/19 and 11/18/20). None of the sampled days recorded any rainfall.

### Water sample processing and sequencing

In total, 10 water samples were analysed to study the ARG presence in the prokaryotic and viral fractions (Supplementary Table S1). Three different analyses were performed: in silico characterization of the ARG using metagenomics, the study of two selected ARGs (*sul2* and *tetW*) by dPCR in water samples from hospital, WWTP input, WWTP output, and seawater, and the search for the ARG presence in RNA and DNA in viruses from waters of the WWTP input and output.

Water samples from hospital (10 mL), WWTP input (10 mL), WWTP output (10 mL), and untreated drinking waters (106 mL) were filtered using a 0,2 μm filter (Isopore Membrane Filters, Ref. GTTP02500). Those filters were used to perform the DNA extraction from the prokaryotic fraction. Prokaryotic DNA fraction from the seawater sample used in this study was obtained as described [29]. The acid nucleic extraction was performed using MasterPure Complete DNA and RNA purification (Epicentre, Ref. MC85200) for all prokaryotic samples except the untreated drinking water, which was processed with DNAeasy PowerSoil Pro (Qiagen, Ref. 47,014) as recommended by the manufacturer. WWTP output samples were the control samples from the Maestre-Carballa (2019) study that were processed as indicated in the paper [24].

Regarding the viral fraction (< 0,2 μm), samples from the input and output of the WWTP and hospital were filtered through a 0,2 μm filter with a syringe (PES membrane, Millipore). The filtered elute water (used volumes at Supplementary Table S1) was then concentrated with tangential ultrafiltration using Vivaflow (100 KDa; Sartorius, Ref. VF20P4) until a final volume of 19 mL, which was again filtered by 0,2 μm as above. The filtered sample was concentrated using Amicon ultra-15 (100 KDa; Millipore, Ref. UFC910008) to a final volume of 200 µL. For the viral ARN samples, 5 L of WWTP input and 5 L of WWTP output were sampled. Both were centrifuged at 10,000 g for 20 min (4 °C) and the pellet was discarded. The supernatant free of cells was ultraconcentrated employing Vivaflow (100 KDa; Sartorius, Ref. VF20P4) until a final volume of 30 mL. 3% beef extract (Sigma-Aldrich, Ref. B4888-50G) and NaNO3 (2 M final concentration; Scharlau, Ref. SO05010500) were added and the pH was adjusted to 5,5. The mix was incubated for 30 min at room temperature and the pellet was eliminated after 10 min of centrifugation (2500 g). The supernatant pH was then adjusted to 7.5. Polyethylene glycol 6000 (PEG; Sigma-Aldrich, Ref. 81253-250G) at 15% and NaCl (2%; Fisher, Ref. BB358-1) were added to precipitate the viruses and the mix was incubated at 4 °C overnight. Viruses were obtained in the pellet after centrifugation (10,000 g, 30 min, 4 °C) and resuspended in 10 mL of PBS (pH 7.4) (Adriaenssens et al., 2018). SYBR Gold (ThermoFisher Scientific, Ref. S11494) was used to confirm the lack of bacteria in viral fraction samples, and then they were concentrated with Amicon ultra-15 (100 KDa; Millipore, Ref. UFC910008) until a final volume of 200 µL.

Free DNA from the viral samples concentrated with Amicon ultra-15 (100 KDa; Millipore, Ref. UFC910008) was eliminated using 1 ul of Turbo DNase I (Invitrogen, Lituania, Ref. AM107) and 20 µL of DNase buffer at 37 °C. After 30 min, 4.22 µL of RNase were added and both enzymes were deactivated 30 min later at 72 °C for 10 min. To ensure the proper nucleic acids liberation and protein digestion, the sample was treated with 1% of proteinase K (50 µg/µl, Epicentre, Ref. MPRK092) and 20 ul of TE 10X at 65 °C for one hour while shaking. The enzyme was inactivated at 4 °C for 5 min. The protocol Qiamp MinElute Virus Spin Kit (QIAgen, Ref. 53,704) was used to extract the nucleic acids. For the RNA samples, instead of the RNA carrier provided in the kit, 21.25 µL of glycogen was added (20 mg/mL; Thermo Scientific, Ref. R0551) to 200 µL of the AL buffer. DNA and RNA concentrations were measured with Qubit HS dsDNA (Thermo Fisher Scientific, Ref. Q32854) and HS RNA (Thermo Fisher Scientific, Ref. Q32852) respectively. In the RNA samples, the DNA was digested with Turbo DNase I (Invitrogen, Lithuania, Ref. AM107) for 45 min at 37 °C to increase the ratio RNA:DNA in the sequencing process.

Metagenomic DNA library preps were carried out with Nextera XT DNA library kit (Illumina, Ref. FC-131-1024) according to the manufacturer´s protocol. All samples were sequenced in a MiSeq Illumina sequencer (2 × 300), except the untreated drinking water sample that was sequenced in a HiSeq X sequencer (2 × 150). Sequencing was performed by Macrogen company (Seul, Rep. of Korea).

Metagenomic RNA libraries from the prokaryotic fraction were performed with Illumina stranded total RNA Prep, in which a step of rRNA depletion was included (Ribo-Zero Plus; Illumina, Ref. 20,040,525). For the RNA viral fraction, the metagenomic library was done using TruSeq Stranded mRNA kit (Illumina, Ref. 20,020,594) avoiding the step where mRNA is purified, allowing us to analyse all RNA present in the sample. All RNA samples were sequenced in a HiSeq 2500 (2 × 125). Sequencing was performed at the Genomics Center of CRG (Barcelona, Spain).

### Bioinformatic analysis

Raw data from water samples was quality-filtered using Trimmomatic 0.36 [3] (SLIDINGWINDOW:4:20, MINLEN:36). Then, the filtered and clean reads were assembled using SPAdes [2] (-meta), and only the obtained contigs > 500 pb were considered for further analysis. From those contigs, ORFs were predicted using Prodigal program [15]. ARGs were annotated by comparing the ARG databases ARG_ANNOT [13], RESFAMS [11], and CARD [16] with both the assembled (ORFs) and unassembled data (reads) using blast. Only the best-hits with a bit-score ≥ 70, e-value < 10^− 5^, and identities ≥ 50% or ≥ 90% (both thresholds were initially compared selecting later the cut-off of ≥ 90% as likely the most reliable) were considered as potential ARG. The housekeeping genes present in the used ARGs databases were not considered in our metagenomic analysis due to the difficulty of determining if they were *bona fide* ARGs using our thresholds [25] According to the database CARD [16] or the nr database (NCBI), the detected ARG were grouped by the antibiotic they confer resistance to. ARG abundance and normalization were estimated by dividing the total number of ARGs per Gb of metagenome (assembled or unassembled) and if necessary, by volume sample as well. Estimation of shared ARGs in the analyzed samples was carried out using a Venn diagram from the bioinformatics UGent webpage (https://bioinformatics.psb.ugent.be/cgi-bin/liste/Venn/calculate_venn.htpl). Those contigs that presented two or more ARGs that conferred resistance to at least two different antibiotic classes were classified as multi-resistant.

In addition to the physical separation of prokaryotic (> 0,2 μm) and viral fractions (< 0,2 μm), bioinformatics was also used to specifically detect viral contigs as follows. All assembled metagenomes were analysed with VirSorter2 [12], which identified viral contigs (max.score ≥ 0.9) in the > 0,2 μm fraction and < 0,2 μm fraction, that were grouped in the hereafter named “putative viral fraction”. Contigs without detecting viral proteins were grouped in the prokaryotic fraction, whose origin could be DNase-resistant DNA, DNA fragments in vesicles, or viruses that were not detected [24]. The contigs in which at least one ARG was detected (through blastp analysis of Open Reading Frames against ARGs databases, as explained above) were annotated using Kaiju [30]. The annotation involved a comparison with the database nr_euk (-E 0.00001, greedy mode, 12/22/23).

To identify viral RNA contigs, the program hmmsearch (hmmer.org) was used to search for RNA-dependent RNA polymerase (RdRP) profiles. The RdRP hidden Markov models (HMMs) used were downloaded from Pfam [31], from other RNA viruses’ papers [5, 42], or generated by HMMER 3.2.2 (hmmer.org) using the sequences obtained from IMG/VR [37].

To validate our results regarding ARG in viral contigs, we analysed the IMG/VR v4 database, which is the most comprehensive database to date and contains high confidence viral contigs from DNA and RNA [6], and also the viral dataset of Atlantic Ocean RNA viruses [40]. Then, we used the same pipeline described above to study the presence of ARG. The taxonomy, host and environment associated to each virus were extracted from the same database [6].

We sought to compare the relative frequency of ARGs (unassembled data; grouped by the drug class they confer resistance to) with the human antibiotic consumption in our country region using Spearman’s correlation coefficient and its significance with a t-student test using R program v. 4.1.2 (R Core Team, 2007). The antibiotic consumption data for the public hospitals and the whole community (including public hospitals and the private sector) was obtained from the PRAN webpage (Spanish Action Plan on Antimicrobial Resistance; https://www.resistenciaantibioticos.es/es/publicaciones/spanish-action-plan-antimicrobial-resistance) as defined daily doses (DDD) of common antibiotics per 1000 inhabitants and day from the Comunidad Valenciana region (year 2019).

### Digital PCR for *tetW* and*sul2*

Absolute abundances of two abundant and ubiquitous antibiotic resistance genes (s*ul2* and *tetW*) in the analyzed water samples were studied by dPCR. For dPCR primers and probes design, PrimerQuest Tool (https://eu.idtdna.com/pages/tools/primerquest) was used. To obtain the target sequences for both ARGs, first all hits obtained from the assembled data were clustered (95% identity) with CD-HIT program using default parameters. *TetW* and *sul2* clusters were selected, and contigs containing one of those ARG were aligned with each other and their corresponding ARG entry in the ARG databases (gb|AAL59753.1|ARO:3,000,412|sul2 or AJ222769_gene_p01 for *tetW)* using MAFFT Alignment v.7.222 [19] available in Genious v. 9.1.3 program. This program generated the consensus sequences, which were used as target sequences to design the *sul2* and *tetW* primers and dPCR probes.

The primer specificity was checked using primer-Blast (NCBI) against the nr database (NCBI). The primer sequences were *tetW*_F (5’->3’) TCCAGTGGCACAGATGTAAAG and *tetW*_R (5’->3’) CTTTAGCGGAGATCACCAAGAT. Regarding *sul2*, the sequences were *sul2*_F (5’->3’) ATGCGCGCGTCAAAGAA and *sul2*_R (5’->3’) ATCTGCCAAACTCGTCGTTATG. Probe sequences were for *sul2* 5’/6-FAM/CG CAA TGT G/ZEN/A TCC ATG ATG TCG CC/3IABkFQ/3’ and for *tetW* 5’/6-FAM/AG GTG TAC C/ZEN/G CTC TTT GGC TGT TT/3IABkFQ/3’.

Primers were checked with a PCR reaction with the same samples that were used to design them. The reaction mixture included 18,15 µL mili-Q water, 1 µL of each primer (10 µM), 0,75 µL of MgCl2 (50 Mm; Invitrogen, Ref. Y02016), 2,5 µL of Buffer 10x (Invitrogen, Ref. Y020228), 0,5 µL of dNTPs 10 mM (Thermo Fisher Scientific, Ref. 10,297,018), 0,1 µL of Taq polymerase (Thermo Fisher Scientific, Ref. 10,342,020) and 1 µL of sample. PCR reaction conditions were: 94 °C for 5 min, 35 cycles of 94 °C for 45 s, 55 °C for 45 s, and 72 °C for a minute and a half. A final extension step was included at 72 °C for two minutes and then held at 4 °C. The PCR products were observed with an electrophoresis gel (Agarose 2%, TBE 1X) and sequenced by Sanger (ABI PRISM 310 Genetic Analyzer. Applied Biosystems) in the Research Technical Services of the University of Alicante.

Digital PCR for *sul2* and *tetW* were performed in a 14,5 µL mixture reaction that included 7,25 µL of MasterMix QuantStudio 3D DIGITAL PCR V2 MMX (ThermoFisher Scientific, Ref. APPA26316), 4,14 µL of mili-Q water, 0,63 µL of each primer (10 µM), 0,35 µL of the probe (10 µM), 2,5 µL of MgCl2 (50 Mm) and 1 µL of the water sample or 1 µL of mQ water for the negative control.

The dPCR mix was loaded into a chip QuantStudio 3D DCPR V2 20 K CHIP (12-PACK, ThermoFisher Scientific, Ref. A26316). The dPCR conditions were: 95 °C for 10 min, 30 cycles of 95 °C for 20 s, 55 °C for 45 s, and 60 °C for a minute. Another phase of 60 °C for 2 min and held at 4 °C. Chips were incubated in dark conditions and room temperature before reading them with QuantStudio™ 3D Digital PCR Instrument (ThermoFisher Scientific, Ref. 4,489,084). The obtained data were analysed with QuantStudio™ 3D AnalysisSuite™ software (ThermoFisher Scientific). The quantification of each ARG was calculated by dividing the number of copies of *tetW* or *sul2* by the ng of DNA from the sample obtained. Replicates and serial dilution DNA samples were included. Before the dPCR, a qPCR was conducted with the same conditions as the dPCR, and the product was run in an electrophoresis gel (TBE, 2% agarose) to check the primer’s performance and accuracy of the primers and probes.

**Fig. 2:**
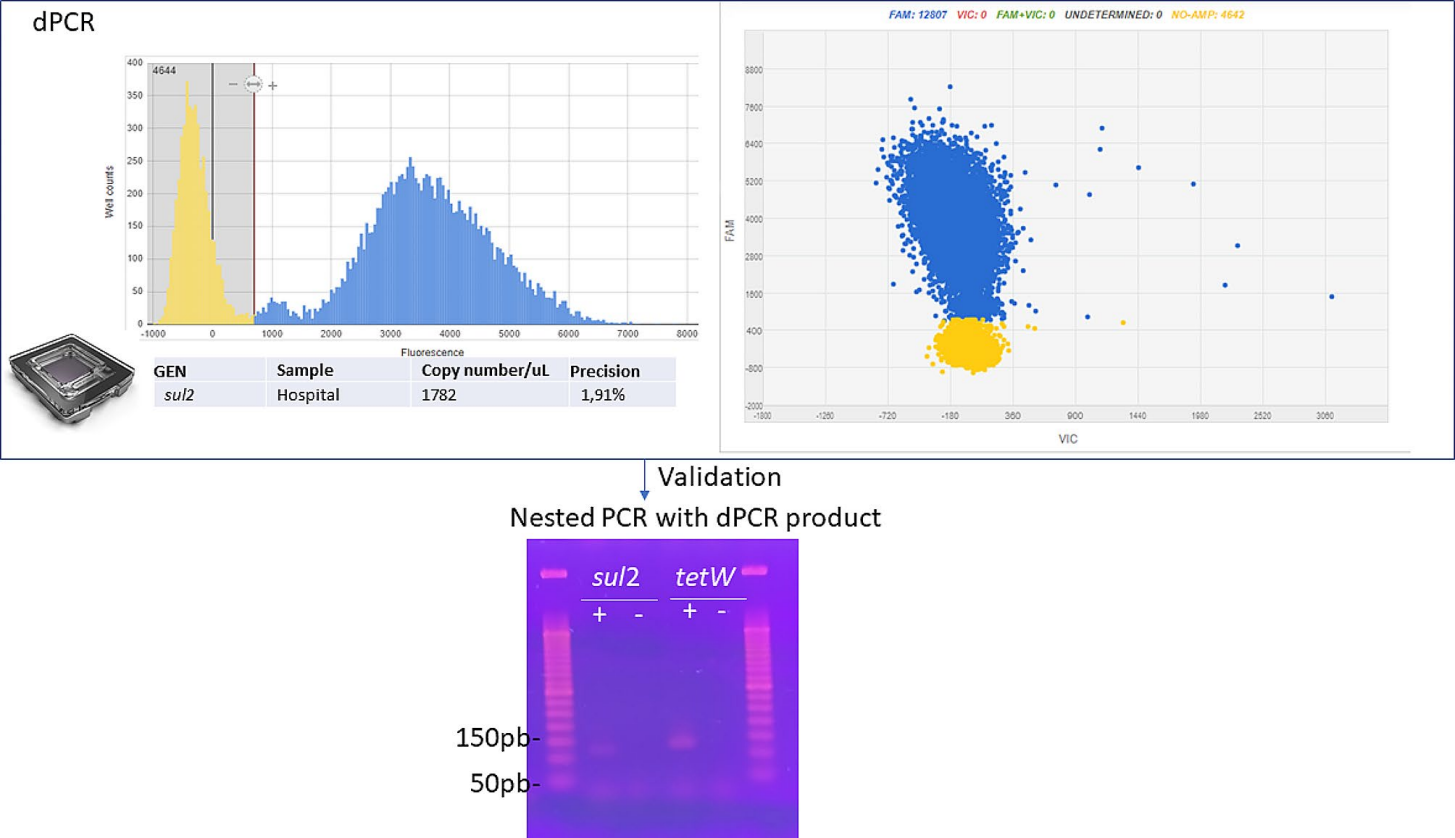
dPCR of *tetW *and *sul*2 antibiotic resistance genes. dPCR primers and probes designed for the ARGs *tetW* and *sul2* were first tested by qPCR (same conditions as the dPCR) in the Hospital wastewater sample, and the PCR products were observed in an electrophoresis gel. Then, a chip-based dPCR was run for both genes and different water samples. Only dPCR results with precision ≤ 10% were considered for our results as recommended for dPCR standards, similarly to qPCR best practices. To verify the proper amplification of the dPCR product, a second PCR (nested PCR) was run with the primers, and the product was analyzed by gel electrophoresis (TBE, 2%) to ensure the expected size of dPCR product. Ladder used: Gene Ruler 1 kb+. This confirms that the detection signal obtained from probes using during dPCR was fully specific as shown in the histogram and dot plot of dPCR detection of targeted genes (blue color)

To verify the proper ARG amplification during the dPCR reaction, the obtained dPCR product was later recovered from the amplified dPCR chip and used as a template in a PCR reaction with the same conditions as above for the dPCR (21,25 µL of MasterMix QuantStudio 3D DIGITAL PCR V2 MMX, ThermoFisher Scientific, Ref. APPA26316, 1 µL of each primer (10 µM), 0,75 µL of MgCl2 (50 Mm; Invitrogen, Ref. Y02016) and 1 µL of the dPCR product). Finally, the PCR product wads analyzed in an electrophoresis gel (TBE, 2% agarose; Fig. 2).

The comparison of the quantification through dPCR and metagenomics for both ARG was performed by contrasting the number of copies of each ARG per ng of DNA with the number of ARG hits (identity ≥ 90%, bit-score ≥ 70 and e-value  ≤ 10^− 5^ with the ARG databases) for both assembled and unassembled data.

## Results

### City-scale resistome

**Fig. 3:**
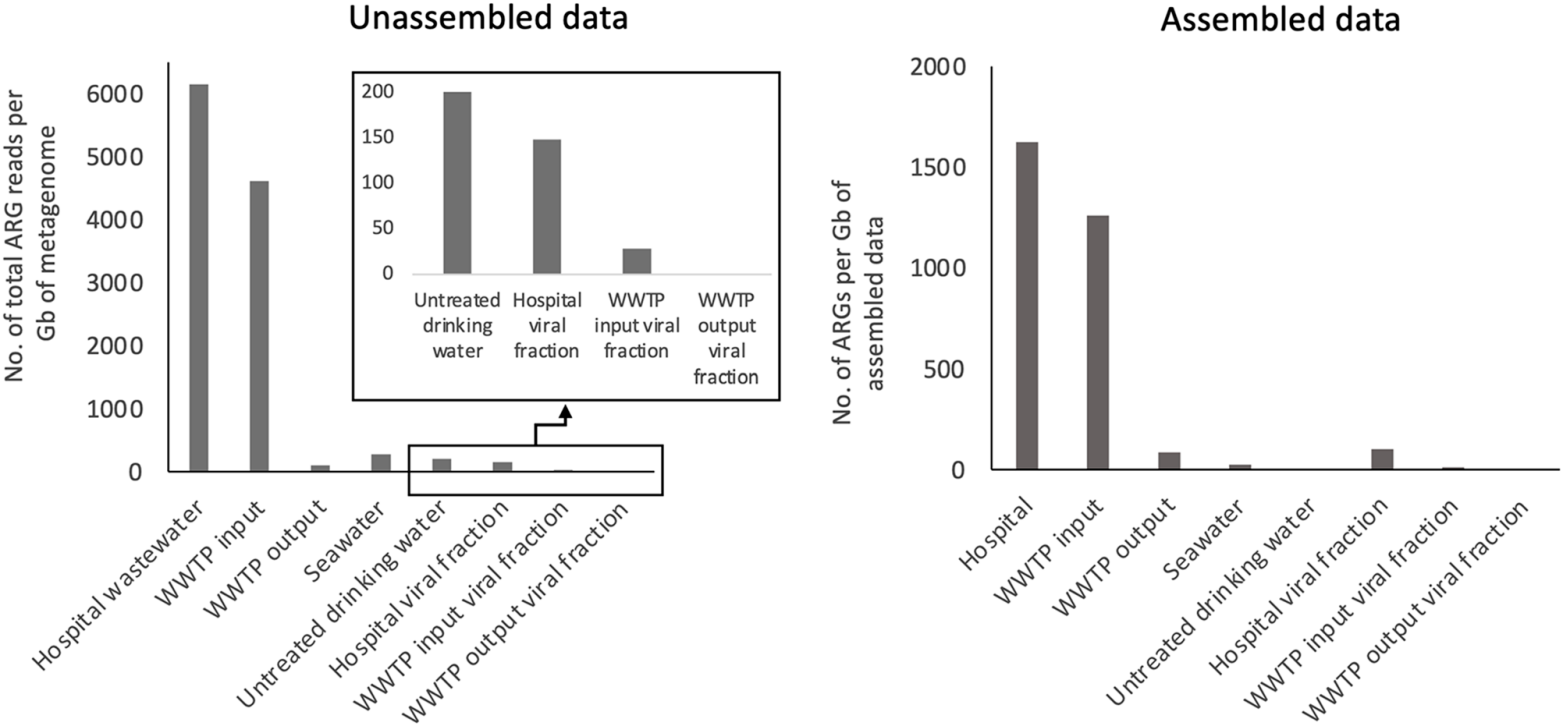
Relative abundance of ARG obtained by metagenomics in the different water samples of Alicante city. The abundance of ARG in each water sample was studied through metagenomics for both unassembled and assembled data. Hospital wastewater and WWTP input had the highest abundance of ARGs. For the assembled fraction, all contigs from the < 0,2 μm and ≥ 0,2 μm fraction in each sample that were classified as viral according to VirSorter (see methods), were grouped in the category putative viral fraction

**Fig. 4:**
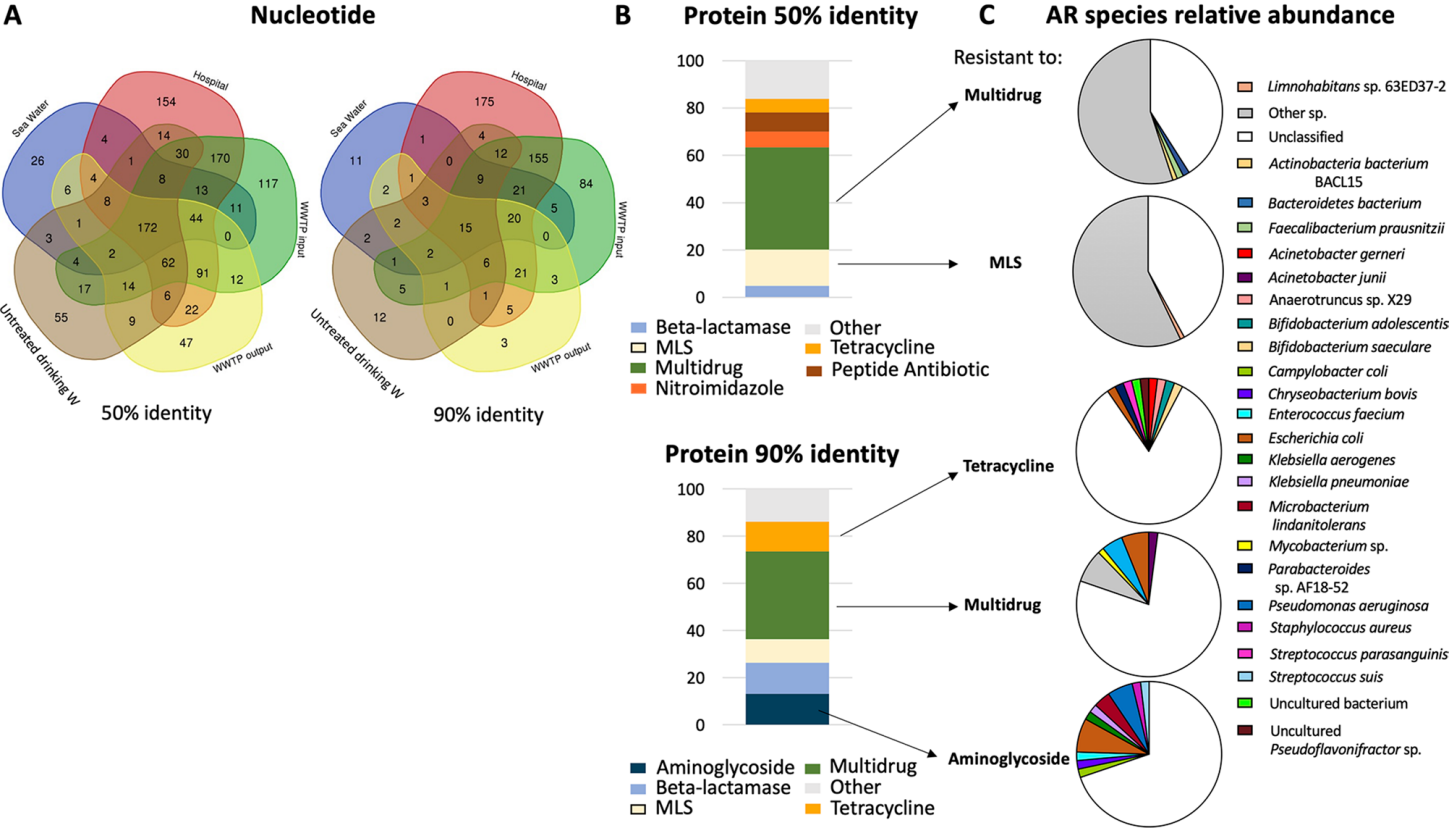
ARGs distribution, relative abundance, and antibiotic resitant bacteria in the different water samples of Alicante. Number of ARGs shared by the different water samples: seawater (blue), Hospital (red), WWTP input (green), WWTP output (yellow), and untreated drinking water (brown) (**A**). For assembled data, different categories of ARG (conferring resistance to multidrug, MLS, tetracycline, and aminoglycoside) were found to be more frequent in Alicante’s water samples at two different protein identity thresholds (≥ 50 and ≥ 90%) (**B**). For assembled contigs, identification of the most abundant bacteria (> 1%) present in the waters of Alicante that had (at least) one ARG that belonged to the most common categories found in the waters of Alicante (**C**)

First, resistome analysis through metagenomics was conducted on a city-scale water distribution system, encompassing samples from untreated drinking water supplying Alicante city, various sewage and wastewater samples, and seawater collected near the discharge point of treated wastewater from one of the largest WWTPs (Fig. 1). The largest ARG abundance and richness (i.e. thousands of ARGs per Gb of metagenome) were detected in hospital wastewater and WWTP input (Fig. 3; Supplementary Table S2) with a large diversity of ARGs conferring resistance to multiple common antibiotics (e.g. multi-drug, beta-lactamases or macrolide-lincosamide-streptogramin; Fig. 4 and Supplementary Figs. S1 and S2). Although metagenomics identified common antibiotic-resistant and multiresistant bacteria, a large fraction remained unclassified or ambiguous at the genus level (Fig. 4C), suggesting a large diversity of uncultured and environmental bacteria yet to be discovered hosting ARGs. The lowest richness and ARG abundance -one order of magnitude lower than hospital sewage sample- was found for the untreated drinking water, treated wastewater, and seawater (Fig. 3). Remarkably, the analyzed viral fractions, either DNA or RNA viruses (hospital wastewater and WWTP input and output), did not seem to represent a major threat for ARG dispersion (Fig. 3 and S3) since the detection of *bona fide* viral contigs hosting ARGs (identity ≥ 90%, bit-score ≥ 70 and e-value ≤ 10^− 5^) was extremely infrequent with up to three order of magnitude lower ARG abundance than the sewage prokaryotic fraction. Finally, the antibiotic consumption DDD of common antibiotics per 1000 inhabitants and day did not show a correlation with the ARG relative frequency found in the unassembled data of Alicante’s waters (p-value > 0.5; Supplementary Table S3).

### Digital PCR vs. metagenomics: surveillance and monitoring of two global ARGs dispersed throughout water system

**Fig. 5:**
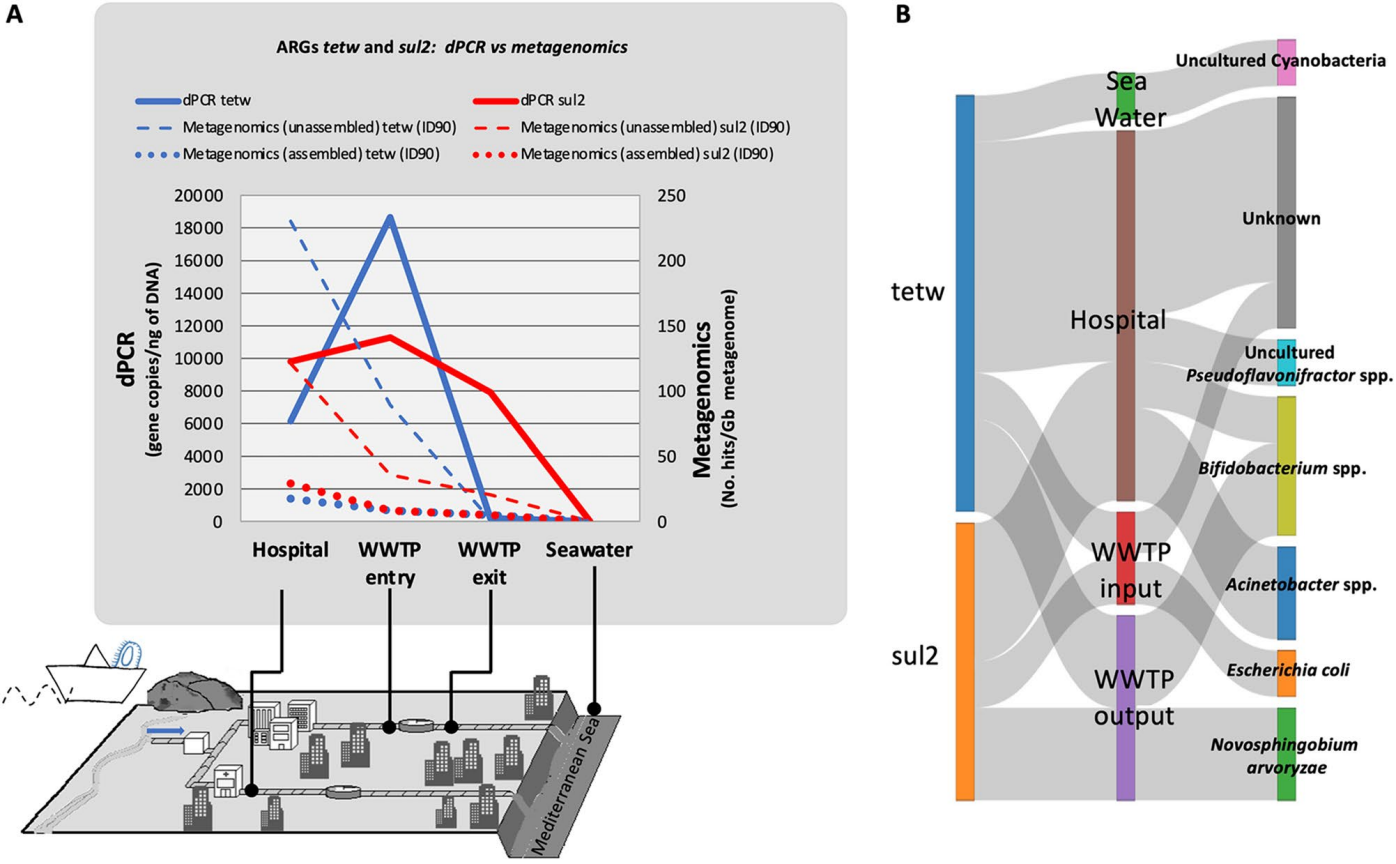
Citi-scale surveillance of the ARGs *tetW* and *sul2* by metagenomics and digital PCR. The relative and absolute abundances of *tetW* (blue) and *sul2* (red) was studied using dPCR (gene copies per ng of DNA) and metagenomics (unassembled and assembled data; No of hits per Gb) for four water samples obtained from the hospital, seawater, and wastewater from the WWTP input and output of the city of Alicante (**A**). Error bars depict standard deviations. Identification of antibiotic resistant bacteria carrying *tetW* or *sul2* in the analyzed samples (**B**)

According to the global resistome analysis, 15 different ARGs were common and present in all the analyzed samples at the city-scale level (Fig. 4A). Amongst the list of ARG city-core, genes *sul2* and *tetW* were selected based on their importance and worldwide distribution [14, 44] for side-by-side comparison by digital PCR and metagenomics (Fig. 5). Overall, the absolute abundances of selected genes by dPCR ranged from 6,000 to 18,600 gene copies per ng of DNA for sewage samples (e.g. hospital wastewater and WWTP input) to only 6.8 gene copies/ng of DNA for the seawater sample collected nearby the WWTP discharging point (Fig. 5; Supplementary Table S4). When conversing these values into absolute number of gene copies of *sul2* and *tetW* per mL of sample (r^2^ correlation of 0.89–0.9; Supplementary Fig. S4), overall data ranged from hundred thousand or thousands copy genes per mL in sewage (maximum value of 6.86 × 10^7^ copies/mL for *tetW* gene from the WWTP input sample) up to less than 5 copies per mL in seawater for both genes (Supplementary Fig. S5; Supplementary Table S4). The absolute abundance after wastewater treatment was still high for *sul2* gene, being carried by different genera, such as *Bifidobacterium* and *Novosphingobium* (Fig. 5B). However, as shown in Fig. 5B, these ARG were not detected in any marine bacteria.

Overall, a similar tendency in the abundance of *sul2* and *tetW* from dPCR was obtained by metagenomics (either assembled or unassembled data; see Fig. 5 and Supplementary Table S4), with higher abundances (36–230 ARG hits per Gb of metagenome; unassembled data) in hospital sewage and WWTP input, and significantly lower values for the seawater sample and WWTP output (0.06-20 ARG hits per Gb of unassembled data). When looking at the metagenomic assembled data, *sul2* was not detected in the assembled contigs of the seawater sample whereas it was detected in the unassembled and dPCR seawater data. This only highlights that metagenomic assembly is imperfect [23]. Some discrepancies were observed with dPCR data, since the highest abundance obtained by metagenomics for *sul2* and *tetW* was observed for the hospital sewage (Supplementary Table S4), while by dPCR, maximum values were obtained for the WWTP input sample instead (Fig. 5). Although a similar abundance trend was observed between samples from both approaches, direct conversion and correlation of dPCR absolute abundance units (gene copies/ng or mL of sample) into metagenomic abundance units (no. of ARG hits from assembled or unassembled data) showed r^2^ values below 0.4, suggesting that several intrinsic factors from each technology might introduce variability that precludes a robust correlation.

The most commonly resistant identified bacteria hosting the studied *tetW* and *sul2* genes in sewage and treated wastewater were overall *Escherichia spp., Acinetobacter* spp., *Novosphingobium* spp., *Bifidobacterium* spp., and an uncultured *Pseduoflavonifractor* spp. (Fig. 5). However, particularly for the hospital wastewater, a large fraction of prokaryotes hosting the gene *tetW* remained unidentified. Remarkably, in the seawater sample collected near the WWTP discharging point, we indeed detected an uncultured cyanobacterium hosting *tetW*, which suggests a possible horizontal gene transfer event.

## Discussion

As the study of ARG in environmental samples has gained more attention, several culture-independent methods have been usually applied, such as metagenomics, a non-targeted method that provides a broad overview of ARG in a sample [25, 39], quantitative PCR (qPCR) that screens specific target genes, or more recently digital PCR, which provides some advantages over qPCR to estimate absolute abundances of copy genes [8, 28, 43]. Here, in a step further, we have implemented and compared dPCR and metagenomics to assess the resistome and abundance of two of the most global ARGs, representing to date likely one of the first examples using in parallel two culture-independent methods. In general, both approaches revealed similar trends in ARG abundance and were capable of detecting variations among samples. dPCR proved to be more sensitive and accurate than metagenomics, especially for samples with lower ARG abundance, such as seawater. Recently, similar observations were obtained in a previous survey comparing ARG abundance by qPCR and metagenomics in multiple environmental samples including river samples, which showed the lowest abundances [10]. In our study, there was a discrepancy in the ARG abundance data pattern, since metagenomic data indicated that ARG abundance was highest in the hospital wastewater followed by WWTP input, while the results from dPCR were exactly the opposite. This example likely illustrates the advantages and disadvantages of each technique, since metagenomics provides a broad perspective regardless of mutation rate and diversity of ARG; as long as the targeted ARGs meet the applied bioinformatic thresholds. In contrast, dPCR, like any other PCR based technologies, is more limited by primer specificity, and likely in our study, primers failed to capture some part of the *tetW* and *sul2* genes diversity present in the hospital wastewater (see for instance Supplementary Fig. S6), which showed the highest ARG richness and diversity (Supplementary Fig. 4A).

In the case of metagenomics, detection of ARG from both assembled and unassembled data analyses was performed considering only very strict thresholds (e.g. ≥90% nucleotide sequence identity; see methods), such as the one recently proposed in the ISO-certified genomics workflow, in which cut-offs of ≥90% nucleotide sequence identity representing “exact” or “close matches” were considered and subsequently PCR validated [38]. To date, in general, there is a paucity of highly accurate, reproducible, and standardized bioinformatic tools for ARGs detection, and this is one of the main limiting factors for wider application of metagenomics. In our case, we have used powerful programs well implemented in metagenomic analysis that rely on sequence search similarity [25, 30]. In our study, we have used multiple well-curated and standard ARG databases for a comprehensive analysis widely used in ARG surveillance (see methods), such as the one used in the recently reported ISO-certified genomics workflow [38]. We envisage that a large transnational effort should be executed and independently performed by several laboratories and institutions for a major cross-comparison of metagenomics and quantitative and digital PCR data that will aid assist policymakers and private companies in making better decisions on how to best approach ARG surveillance. In particular, the most interesting and challenging idea in ARG monitoring within the One-Heath perspective would be to develop a standardized methodology of metagenomic and dPCR (or qPCR) in which we would be able to make a direct conversion or correlation of relative abundance units obtained either from assembled or unassembled metagenomic data with absolute abundance units normalized by extracted DNA or volume sample commonly obtained from dPCR. In our study, as shown in the result section, the different methodological biases introduced in each step from each one of the methodologies likely preclude obtaining a good correlation.

ARGs abundance in the input WWTP was lower than the one found at the WWTP output, due to the WWTP treatment, as observed elsewhere [35]. This decrease was observed for both prokaryotic and viral fractions. A substantial portion of microbes harbouring antibiotic resistance genes (ARGs) remained unidentified, pointing out to a considerable diversity of antibiotic resistant bacteria yet to be revealed. Among the ones that could be classified, we found many bacteria related to faecal contamination (i.e. *Escherichia coli*, *Enterococcus faecium*, *C*ampylobacter *coli*), a factor which could largely explain the ARG abundance in polluted sewage environments [18]. Interestedly enough, a comprehensive analysis of ARGs present in the IMG/VR v4 database with over five millions of viral genome and genome fragments (5,621,398 high-confidence viral contigs) showed that only ~ 0.04% of the viral contigs harbour ARG, with being the multidrug resistance the most frequent category (Supplementary Table S5). Most of DNA viruses carrying at least one ARG were classified as Caudoviricetes and Tubulavirales, and the host bacteria were in fact human associated species (Suppl. Table S6).

Given that the wastewaster is a hot-spot for the ARG dispersion [22] and considering the abundance of bacteria that are targeted by RNA viruses such as *Pseudomonas* preyed by *Cystoviridae* [26] or *Escherichia coli* that could be infected by the *Qβ* phage [4] (being the later one of the most prevalent antibiotic resistant bacteria identified in our samples (Fig. 4B)), we sought to explore the presence of ARGs in RNA viruses in our WWTP samples. No ARGs were found in RNA viral contigs in the analyzed wastewater samples. In order to corroborate our data, we explored the presence of ARG in other RNA viral datasets such the ones included at IMG/VR V4 [6], that contains, among others, RiboV1.4 with a total of 378,253 RNA viruses obtained from the study of different metatranscriptomes [33] and also RNA viral contigs from Atlantic ocean recently published (*n* = 2692) [40]. We did not find any ARG in those RNA viruses with the thresholds used. The absence of ARG in RNA viral contigs could be explained by the short genome size of those viruses [7], being unlikely to carry auxiliary metabolic genes. Overall our data suggest that although wastewater viruses could act as a reservoir of ARGs, and in good agreement with other authors [9], they do not seem to represent a major threat for the ARG dispersion, especially after the WWTP treatment which reduces the ARG abundance.

Among the ARGs found in our samples, we selected *sul2* and *tet*W due to their importance, since both are included in a recent reported list of ARGs that pose a worldwide health risk [45]. Unexpectedly *sul2* abundance after treatment was still high (unlike *tet*W), suggesting that a large potential of *sul*2 ARG could have been discharged into natural environments, such as the Mediterranean Sea. However, these ARG do not seem to be later acquired and evolutionary selected by autochthonous marine bacteria (Fig. 5). The only case that we found that points out in that direction is the presence of *tet*W in a cyanobacteria, that could represent a case of horizontal gene transference (HGT). The presence of *tet*W in cyanobacteria has also been found using dPCR in eight water samples nearby the Taihu Lake (China) [41].

## Conclusions

One of the main goals of this study is the comparison of metagenomic and dPCR abundance data for two of the most globally dispersed ARGs: *tetW* and *sul2* providing resistance to widely used antibiotics; sulfonamides and tetracyclines. Different hot spots at a city-scale level were considered, and in general, both techniques were able to show similar abundance tendencies albeit some discrepancies were also observed. dPCR was sensitive enough to detect a few ARG copies and provided an accurate absolute estimation of gene copies per ng of DNA or analyzed volume sample both at very high or low ARG abundance in samples, which is a strength point for further cross-validation studies using dPCR in ARG surveillance. In contrast, our data showed that metagenomics provided a broad coverage of ARG detection but was less sensitive compared to dPCR. Some discrepancies were observed for the ARG abundance pattern in the analyzed samples using both methodologies. In the hospital wastewater sample (highest ARG richness) metagenomics was likely able to capture more ARG diversity and abundance than the one obtained with the used, specific primers for *tetW* gene in dPCR (Supplementary Fig. S6), exemplifying the pros and cons of each technology (e.g., dPCR data is less informative for providing the taxonomic context of microbes carrying the targeted ARGs). Unfortunately, in our study, a direct conversion and correlation of relative units from metagenomics to absolute abundance units from dPCR was not achieved, which highlights that several intrinsic methodological limitations and biases from each one of the techniques have yet to be addressed and preclude a robust and reliable correlation.

### Electronic supplementary material

Below is the link to the electronic supplementary material.

**Supplementary Material 1:** Supplementary Figures

**Supplementary Material 2:** Supplementary Tables

## Data Availability

Sequencing data is deposited under BioProject numbers PRJNA1029559 (water samples) and PRJNA1029899 for WWTP input and output viral samples (RNA and DNA).

## Abbreviations

ARGs: Antibiotic Resistance Genes
MLS: Macrolide-Lincosamide-Streptogramin
WWTP: WasteWater Treatment Plant
