# Supplementary material for: City-scale monitoring of antibiotic resistance genes by digital PCR and metagenomics
Source: Environ Microbiome. 2024 Mar 15;19:16. doi: 10.1186/s40793-024-00557-6 (PMC10943798; doi:10.1186/s40793-024-00557-6)
Supplement: Supplementary file 1 — Supplementary Material 1: Supplementary Figures [file 40793_2024_557_MOESM1_ESM.docx]

**Supplementary Figures**

**City-scale monitoring of antibiotic resistance genes by digital PCR and metagenomics**

Lucia Maestre-Carballa^1,2^, Vicente Navarro^3^, and Manuel Martinez-Garcia^1,2^*

^1^Department of Physiology, Genetics, and Microbiology, University of Alicante, Carretera San Vicente del Raspeig, San Vicente del Raspeig, Alicante, 03690, Spain

^2^Instituto Multidisciplinar para el Estudio del Medio Ramon Margalef, University of Alicante, San Vicente del Raspeig, Alicante, 03690, Spain

^3^Clinical Microbiology and Infectious Disease Unit, Hospital Universitario Vinalopó, Elche, Spain.

**This Supplementary material contains 6 supplementary figures.**


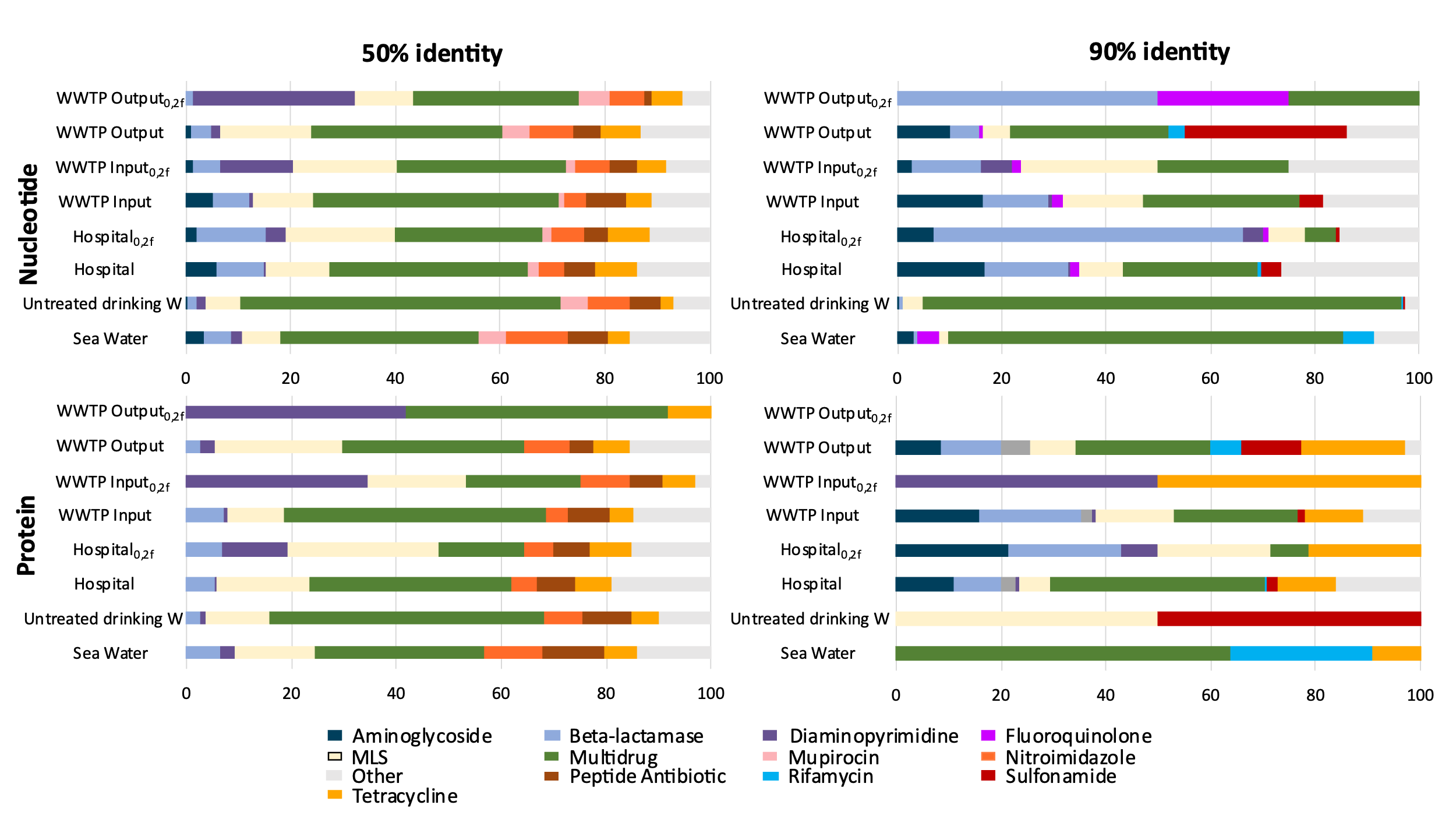


**Supplementary Figure S1. ARGs relative abundance for assembled and unassembled data in water samples of Alicante city.** ARGs were grouped by the class of antibiotic they confer resistance to, for both unassembled data (nucleotide) and assembled data (protein) at two different blast identities (≥50 and ≥ 90%). Only those classes of antibiotics with a relative frequency ≥ 5% were represented. _0,2f_ refers to the 0,2µm-filtered fraction.


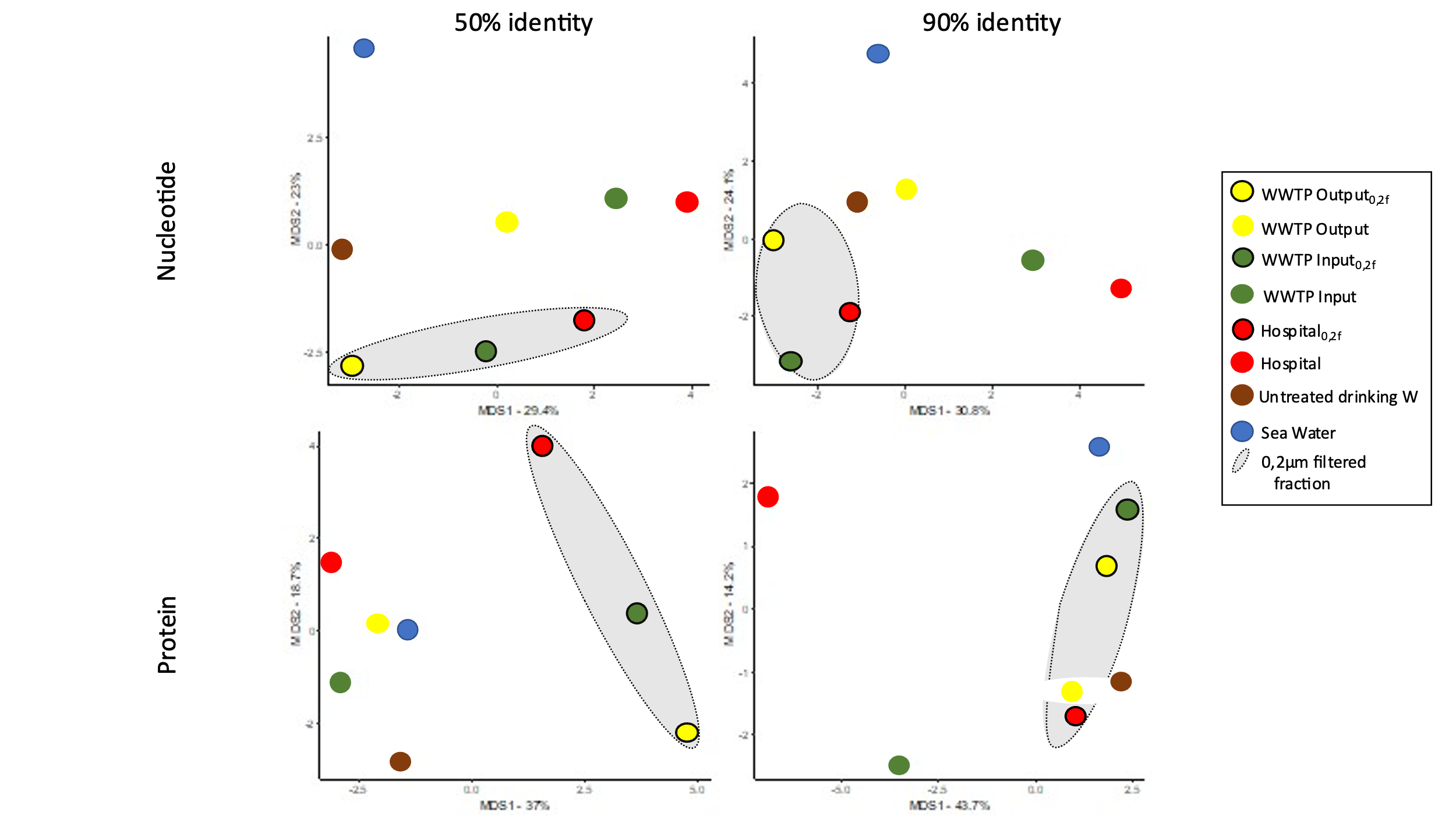


**Supplementary Figure S2. PCoA of the resistome in water samples of Alicante.** PCoA representation of the different water samples of Alicante according to the relative abundance of ARG found for both the assembled (protein) and unassembled (nucleotide) data at two different blast identities (≥50 and ≥90%). 0,2µm-filtered fractions (_0,2f_) are highlighted with a black circle and a grey area.


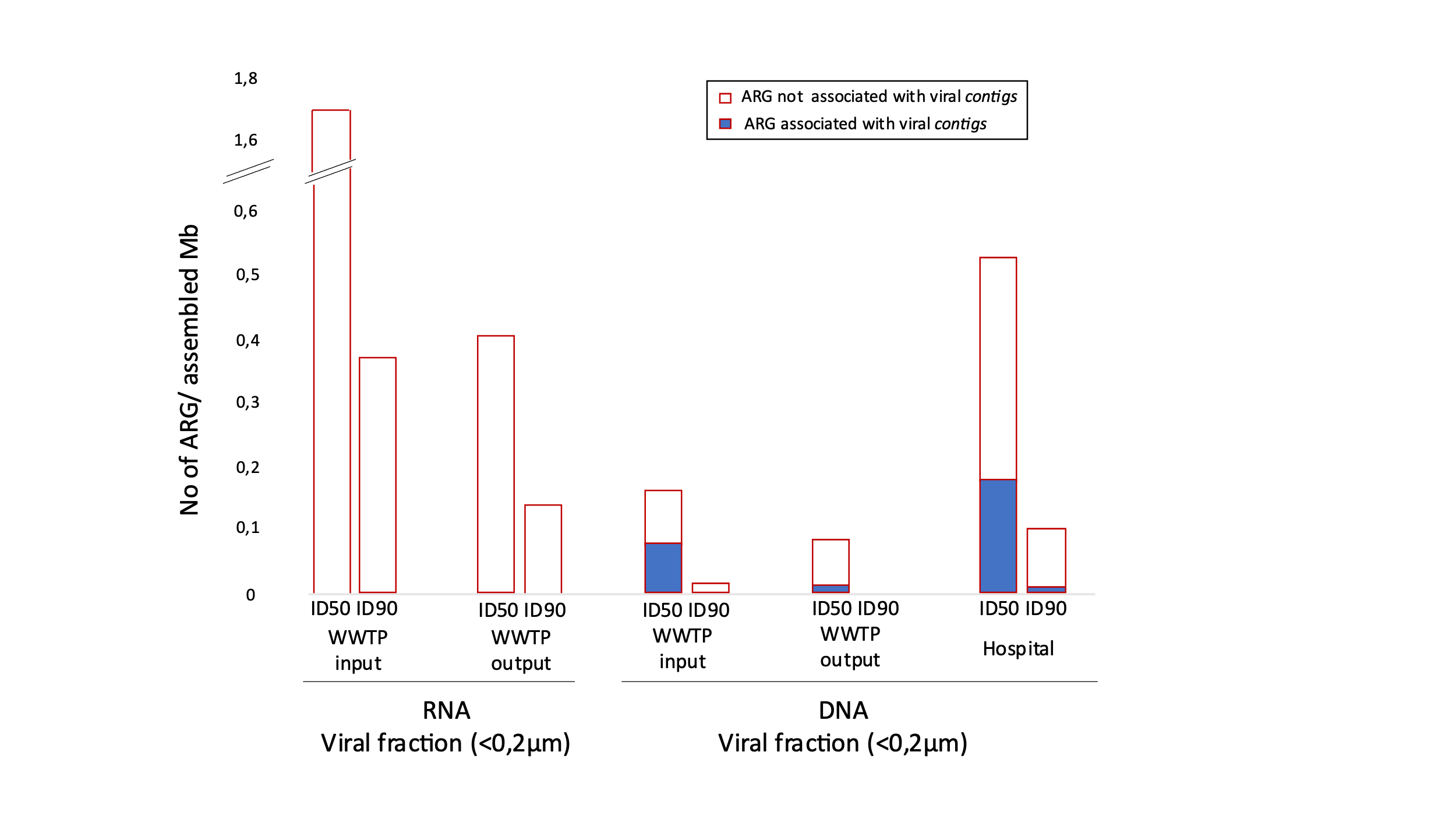


**Supplementary Figure S3. Comparison of ARGs found in RNA and DNA viral fraction.**  Abundance of ARGs detected in the assembled viral fraction obtained from RNA (WWTP input and output) and DNA (hospital, WWTP input and output) samples. Two different blast protein identities were tested (ID) ≥50% and ≥ 90%. Red bars indicate the abundance of ARGs per assembled Mb; inside them, the blue color indicates ARGs/Mb found in the viral fraction associated in silico with viral contigs. White space indicates ARG/Mb that could not be associated with viral contigs.


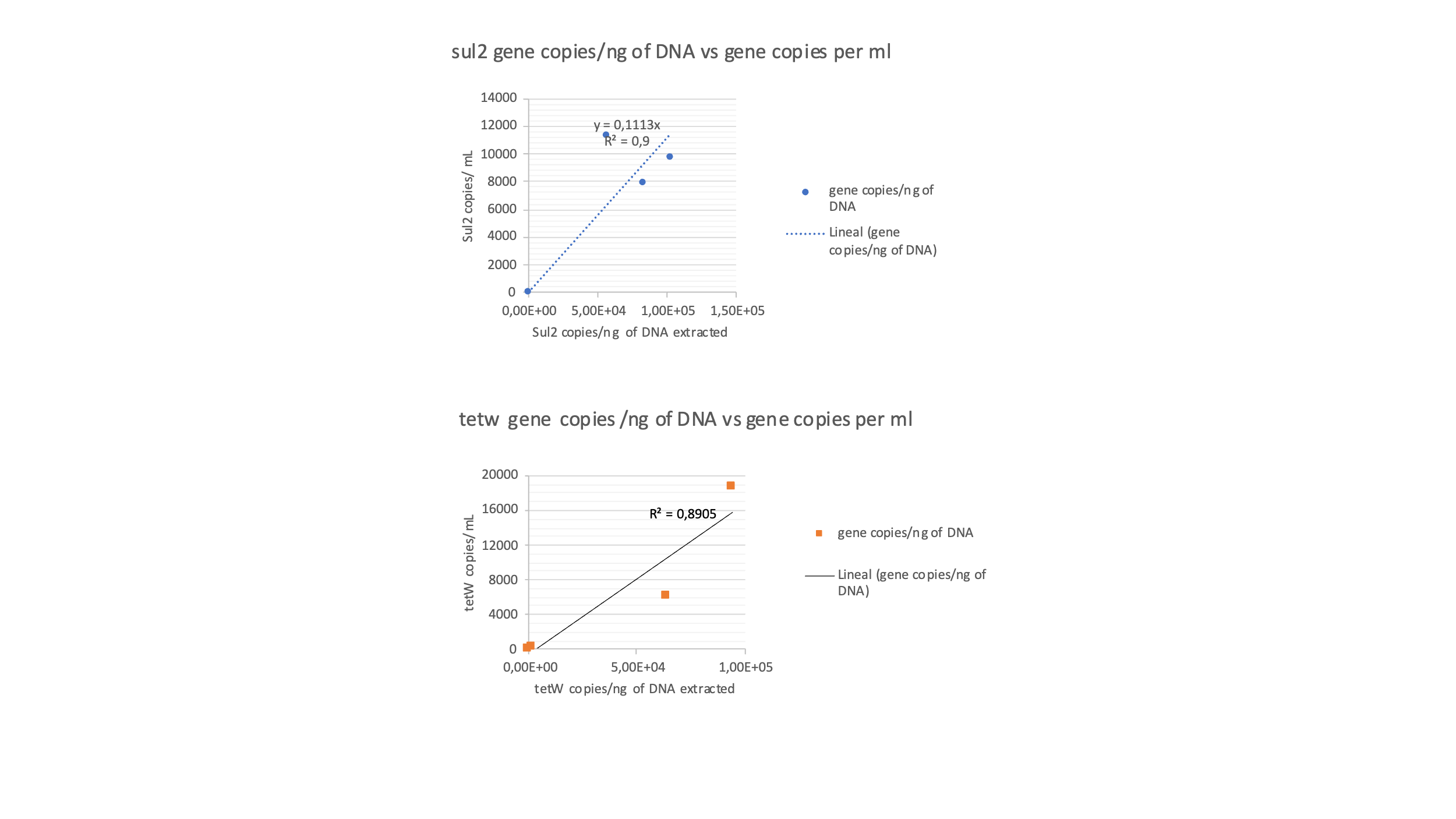


**Supplementary Figure S4. Correlation for the ARG *tetW* and *sul*2 comparing the number of gene copies per ng of DNA versus the number of copies found per mL of sample.** dPCR results (copies of t*etW* or *sul*2) were extrapolated to the number of copies per mL or number of copies per extracted ng of DNA giving a R^2^ ≈0.9 for both genes.


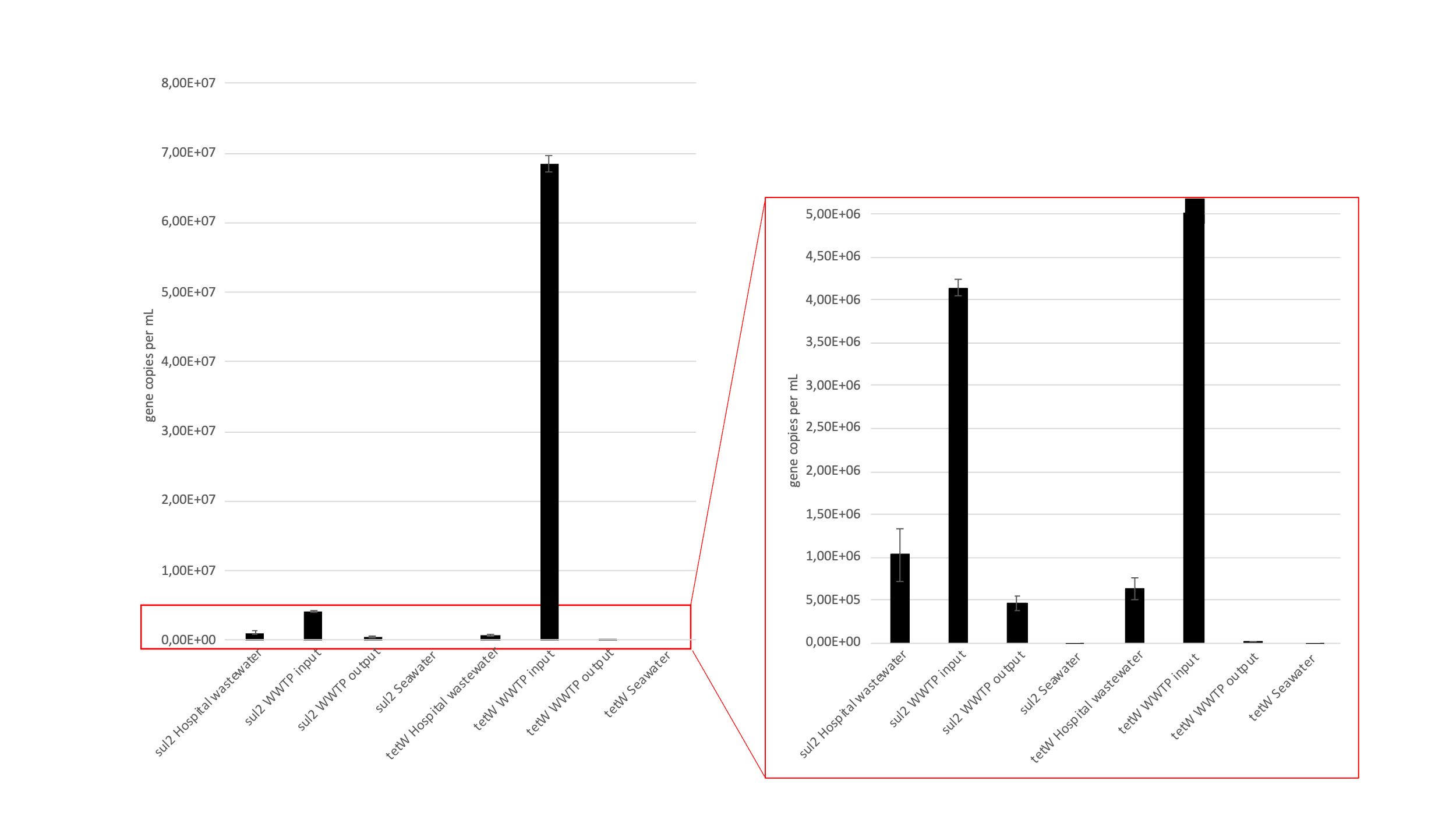


**Supplementary Figure S5. dPCR results for *tetW* and *sul*2 in water samples.** Number of copies of tetW or sul2 per mL of water sample. *TetW* in the WWTP input had the highest number of copies per mL, while the rest of the analyzed samples (hospital, WWTP output and seawater) had less than 4.5X10^6^ copies/ mL.

**
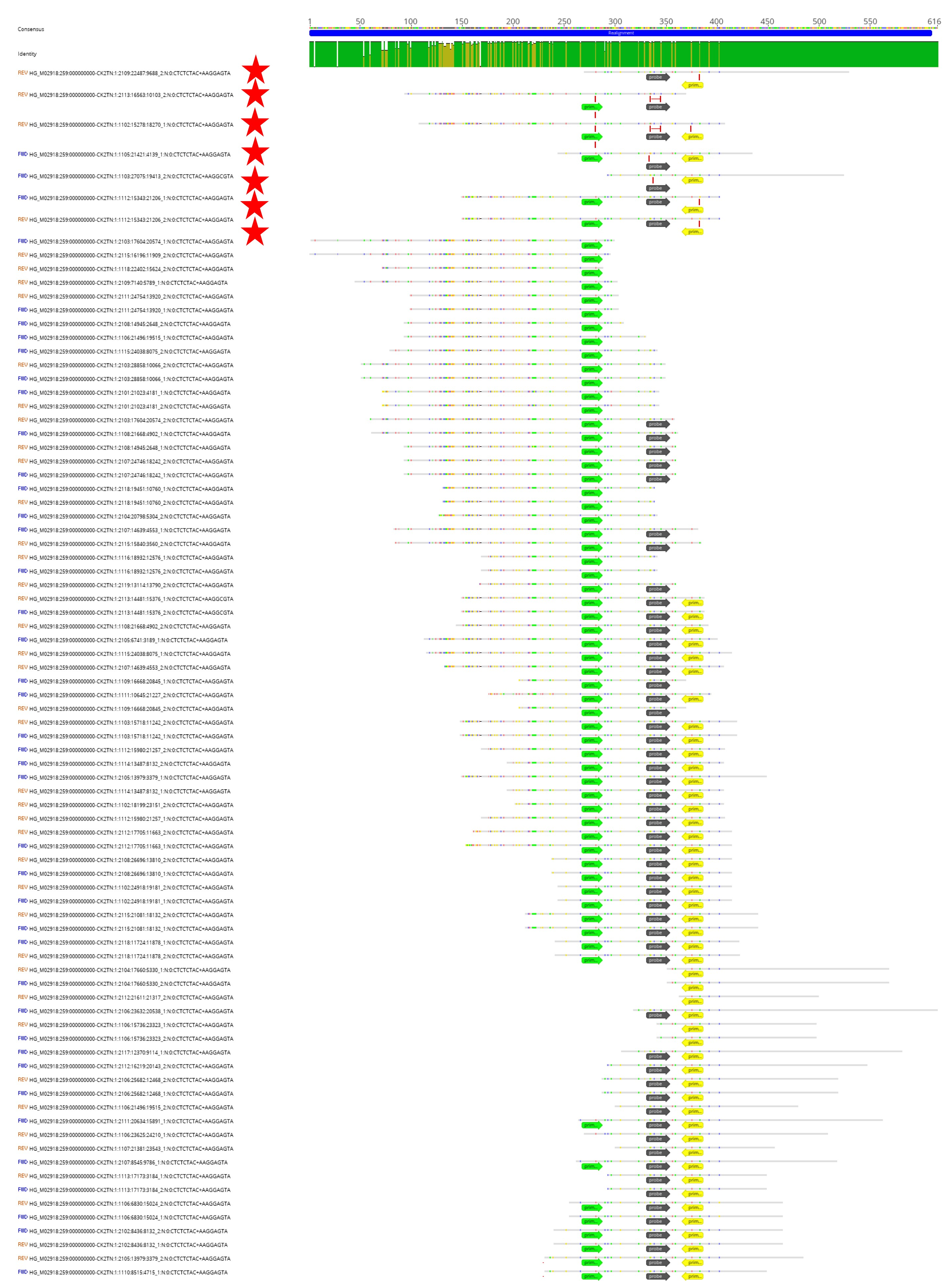
**

**Supplementary Figure S6.** **Alignment of metagenomic reads of *tetW* gene.** The figure shows the detected forward (green), probe (grey), and reverse (yellow) sequences used for dPCR. Note that some reads showed mismatches that might preclude PCR amplification and/or detection by the probe and primers during dPCR (denoted by a red star). All reads aligned showing the overall percentage of nucleotide identities >90%.
